# Evaluation of feed utilization, immune response and disease resistance in striped catfish, *Pangasianodon hypophthalmus* (Sauvage 1878) fed with a novel *Aeromonas hydrophila* biofilm vaccine

**DOI:** 10.1016/j.fsirep.2022.100070

**Published:** 2022-10-08

**Authors:** M.A.A. Mamun, S. Nasren, P.B. Abhiman, S.S. Rathore, K. Rakesh, N.S. Sowndarya, K.S. Ramesh, K.M. Shankar

**Affiliations:** a Laboratory of Aquatic Animal Health Management, Department of Aquaculture, College of Fisheries (KVAFSU), Mangalore 575002, India; b Department of Fish Health Management, Faculty of Fisheries, Sylhet Agricultural University, Sylhet 3100, Bangladesh; c Department of Fish Biology and Genetics, Faculty of Fisheries, Sylhet Agricultural University, Sylhet 3100, Bangladesh; d Veterinary College, Shimoga, Ex-Dean, College of Fisheries (KVAFSU), Mangalore 575002, India

**Keywords:** Biofilm based oral vaccine, Polyclonal antibody, Haemato-biochemical indices, Striped catfish, Gut associated lymphoid tissues, Growth performance

## Abstract

Striped catfish, *Pangasianodon hypophthalmus* was immunized with Biofilm (BF) and Free cell (FC) of *Aeromonas hydrophila* vaccine at 10^10^ CFU g^−1^ for 20 days and monitored for growth parameters, immune responses and disease resistance up to 60 day post vaccination (dpv). Pangasius catfish in the BF vaccinated group had considerably higher growth and feed utilization than the FC vaccinated and unvaccinated groups (*p <* 0.05). Biofilm vaccinated group showed a significant increase (*p <* 0.05) in the mean weight gain (46.91 ± 0.59) than the FC (35.94 ± 0.21) and unvaccinated group (34.92 ± 0.35). The vaccinated fishes were challenged with *A. hydrophila* at 10^7^ CFU/ml. Significant higher relative percentage survival (RPS) was recorded with BF (84.21 ± 1.49%) compared to that with FC (33.33 ± 1.21%). Polyclonal antibody-based ELISA was used to quantify the antibody titre. BF vaccinated group showed significantly higher antibody titer compared to other treatments (*p <* 0.05). Moreover, higher haematological parameters recorded in the present study were differentially stimulated by the oral administration of *A. hydrophila* biofilm vaccine. The mean total protein, albumin, and globulin levels of the BF vaccine groups were significantly higher (*p <* 0.05) than the mean total protein, albumin, and globulin contents of the unvaccinated group. Furthermore, biochemical stress parameters (SGPT, SGOT) in the vaccinated groups showed an incremental trend in the early days of the experimental period. However, the values were significantly lower (*p <* 0.05) in the biofilm group on 20 dpv onwards indicating improved health condition. Vaccinated BF fishes showed gut associated lymphoid tissues (GALT) within the laminar propria of mid gut. But in FC group fishes showed less aggregation of lymphoid cells. The unvaccinated control fish had no lymphoid cell aggregation in their intestines. The findings of the current research suggested that biofilm vaccine has the capability to be one of the potential oral vaccines in striped catfish against *A. hydrophila* infection.

## 1 Introduction

During the last few decades aquaculture has played a vital and steadily rising role in food safety and monetary stability to the world. However, since 2000, global aquaculture now not enjoys the high annual growth and fallen to moderate 5.8% by the period of 2001–2016 [1]. Diseases have been a continuous concern for numerous significant Asian aquaculture producers, notably India. Striped catfish, *Pangasianodon hypophthalmus* (known as pangas/pangasius) an exotic fish species cultured across the country for its faster growth often compromised with bacterial infection. The motile aeromonas septicemia (MAS) and bacillary necrosis in pangasius (BNP) are two main bacterial diseases causing huge economic losses in pangasius farming [2], [3], [4], [5]. Prophylaxis through antibiotic had been an effective strategy in the beginning [6] but antibiotic-resistant pathogenic bacteria are becoming increasingly common, posing a severe threat to human and animal health around the world [7]. Prevention of epidemics is consequently necessary to avoid major economic losses and the development of an appropriate vaccine against the virulent pathogen is therefore imperative [8]. Disease prevention through vaccination and immunostimulation have been shown to be highly beneficial, since the 1980s, when the Norwegian salmon industry proficiently validated the concept of ​​mass vaccination [9]. Vaccination strategy has been established for more than 40 countries against 22 bacterial diseases for more than 17 aquaculture species [10]. The administration of monovalent and polyvalent vaccines in Norwegian salmon industry has not only paved the way for potential solution for prevention of infectious diseases and higher yield but also reduced the burden of antibiotic usage in aquatic environment [11].

The immune protection provided by injection vaccination is significantly superior to that offered by the immersion strategy and the oral approach [12]. However, antigens that persist at the injection site can act as inflammatory reactions, possibly leading to negative side effect [13], growth penalty [14,15] and malfunction of the reproductive organs [16]. Oral vaccines are an attractive alternative to overcome such constraints. Feed based oral immunization is one of the most popular technique among the administered types because of its low cost, easy to handle and can immunize fishes of all size at a time [17].

However, conventional free cell bacterial vaccine provide lower and inharmonious immune response and disease resistance in fish [18] due to acid destruction of the antigen in the stomach / foregut prior to the entering the hind gut where immune induction initiated [19]. Several techniques have been developed and tried to protect the antigens from destruction viz., encapsulated antigen microspheres [20,21], liposome vaccine [22] enteric coated vaccine [23] and artemia [24] for protecting the antigens from enzyme destruction, which many are costly and complex. Against this background, biofilm based oral vaccines look promising because of its cost-effective preparation, non-stressful and mass immunization.

Biofilm based oral vaccine has been conceptualized in our laboratory. *In vitro* biofilm of *A. hydrophila* developed on a substrate (chitin flakes) later inactivated and mixed with fish feed. The biofilm vaccine feed has given significant encouraging results in different models of fish experimented in herbivore [25], omnivore [26], carnivore [27], Nile tilapia [28], Asian seabass [29] and penaeid shrimp [30] in terms of antibody titre and protection upon challenge. However, vaccine efficiency should not rely on protection alone [31] rather its ability on growth performance, blood profile as well as biochemical response to vaccine [16] and those parameters can provide necessary facts to select an ideal antigen for vaccination [32]. So far, there has been no published information on growth performance and gut morphology [33] of fishes fed on biofilm *A. hydrophila* as oral vaccine. Therefore, the present study was designed for the evaluation of *A. hydrophila* biofilm oral vaccine on growth, haemato-biochemical profile and gut histology of striped catfish, *P. hypophthalmus*.

## 2 Materials and methods

### 2.1 Isolation, identification and maintenance of pure culture

A virulent *A. hydrophila* isolated from diseased gold fish was injected and re-isolated from common carp (*Cyprinus carpio*) as described by Mamun et al. [34]. Following confirmation, the bacteria were grown in Trypton Soya Broth (1.5w/v). Cultured broth were transferred to the 50 ml centrifuge tube later harvested as pellet by centrifugation at 10,000 rpm for 10 min. Bacterial sample were preserved with 15% glycerol and 1.5% TSB (w/v). The bacterial suspension was aliquoted into 2 ml micro-centrifuge tubes and preserved at -40 °C for further use.

### 2.2 *In vitro* preparation of *A. hydrophila* biofilm cells

Production of *A. hydrophila* biofilm cells were prepared according to Azad et al.[35]. Media were prepared in 250 ml conical flask bearing TSB (0.225 w/v) and chitin flakes (0.3%) were sterilized by autoclaving. One milliliter (ml) fresh bacterial broth inoculated into the prepared conical flask and agitated for 6 h daily in refrigerated shaker incubator with 120 strokes/min at 37 °C. The biofilm cells were harvested on day four by washing thrice in the same flask with sterile phosphate buffer saline (PBS, pH 7.2) to remove free cells. Before mixing with the feed biofilm cells were heat inactivated (100 °C for 50 min).

### 2.3 *In vitro* preparation of *A. hydrophila* free cells

Planktonic *A. hydrophila* cells were produced according to Azad et al. [35]. Hundred (100) ml media (TSB, 1.5%) autoclaved at 121 ^0^C for 15 min and inoculated with *A. hydrophila*, incubated for overnight at 37 °C. Free cells antigen collected by centrifugation at 10,000 rpm for 10 min and further washed thrice using sterile PBS (pH 7.2). At the end, the bacterial pellets were re-suspended in PBS to achieve higher CFU/ml (10^10^ cells/ml). Free cells or planktonic cells were heat inactivated before incorporating in the feed.

### 2.4 Characterization of biofilms through light microscopy and field emission scanning electron microscope (FE-SEM)

Four-day old biofilm of *A. hydrophila* dislodged from the substrates by vortexing for 5 min and mounted fresh on to the slide, was observed under the phase contrast microscope (Olympus, BX3-25ND25, Japan) and microphotographed.

Biofilm cells were characterized in FE-SEM according to the manufacturer's instruction. BF cells were separated from chitin after vortexing for 3 min, the supernatant, with the cluster of biofilm was centrifuged at 200 rpm for 5 min to remove chitin particles, biofilm in the supernatant was pelletized at 1000 rpm for 10 min and the pellet was resuspended in sterile PBS. A small quantity of prepared biofilm cells were dispersed on a SEM specimen mount. Enough argon was maintained in the chamber so that the vacuum reads 0.08 mbar. Sample was coated with gold (Eiko 1B-3 at 0.15 torr) as appropriate for a specified period of time and current to obtain an acceptable coating. Finally sample was transferred back to the specimen box after coating for analyzing at Carl Zeiss Sigma VP Field Emission Scanning Electron Microscope in the Central Laboratory of DST-PURSE PROGRAMME, Mangalore University.

### 2.5 Incorporation of biofilm and free cells in feed

Biofilm vaccine (BF) as well as free cells (FC) were mixed into the feed as stated by Azad et al. (1999). Different feed ingredients such as fish meal (24%), groundnut oil cake (24%), rice bran (17%), wheat flour (17%) tapioca flour (14%) were collected from local fish market. The ingredients were well sieved, mixed and cooked and cool to ambient temperature. Cod liver oil (3%) vitamins and mineral premix (1%) were added to cooked ingredients followed by incorporation of BF and FC vaccine separately. A basal diet (C) with above components without vaccine antigen was prepared with sterile PBS (pH 7.2). Both BF and FC vaccine were quantified and incorporated in feed at 10^10^ cells g^−1^ of feed at the end. The feed dough was pelletized by hand pelletizer later dried and stored at 4 °C in a refrigerator.

### 2.6 Rearing of pangasius fish stock

Healthy fry of *P. hypophthalmus* were procured from Zonal Agricultural and Horticultural Research Station, Mudigere (13.1378°N 75.6060°E.), Chikkamagaluru, India. Further, the fish were reared for one month to grow into juvenile stage. The fish were fed with dry small pellets, prepared with feed ingredients such as fish meal 24%, groundnut oil cake 24%, rice bran 17%, wheat flour 17%, tapioca 14%, cod liver oil 3%, minerals and vitamins 1%.

### 2.7 Oral vaccination of pangasius catfish

Juvenile *P. hypophthalmus* (12.42 ± 0.14 g) were maintained in cemented rectangular tanks of containing 950 L water. Treatments were assigned into two groups’ biofilm (BF) and free cell (FC) and one control (C) with triplicate. Thirty fishes (juveniles) were released in each replicate. Optimum water quality were maintained by removing of wastes by siphoning (20% water) from the bottom of the tank at every alternate day. Both the vaccinated group (BF and FC) fishes fed vaccine at the dose of 10^10^ g^−1^ of feed. Pangasius juveniles in the control group (C) fed basal diet prepared with PBS. The vaccinated and basal feed was given at 3% of the body weight at 9.00 and 17.00 h for 20 days and the complete acceptance of feed was monitored each day. Wastes were siphoned from the bottom of the tanks once in day and subsequently added 20% water. The water quality parameters such as water temperature, pH, DO and ammonia were 22.5–27.4 °C, 6.9–7.8, 6.5–7 ppm and 0.04–0.06 ppm, respectively throughout the experimental period. After 20 days of trial, vaccinated feed were completely withdrawn and control feed was given till the experimental period.

Upon completion of 20 days of oral vaccination, three fish from each treatment and control were anesthetized (1 ppm, Clove Oil) and bled from their caudal vein of fish using disposable syringes (2 ml) flushed with heparin (Sigma, UK). Blood sample were collected on 0, 10, 20, 30, 40, 50 and 60 dpv. Harvested blood sample separated into two aliquots, one for CBC (Complete Blood Count), and the other sample for serum collection. Blood was centrifuged at 10,000 rpm for 10 min and sera collected from 3 fish per treatment were accumulated and stored at -40 °C for further record.

### 2.8 Estimation of growth parameters

The fishes were sampled at every 15 days to analyze the growth parameters. The parameters such as weight gain, % weight gain, specific growth rate (SGR), feed conversion ratio (FCR), protein efficiency ratio (FCR), average daily gain (ADG), feeding rate (FR) and survival rate were determined according to Rathore et al. [36].

### 2.9 Protection upon challenge with *A. hydrophila*

#### 2.9.1 Estimation of lethal dose_50_ (LD_50_) of *A. hydrophila*

*A. hydrophila* isolate was tested for pathogenicity in fingerlings of *P. hypophthalmus* (15 ± 5.50 g) were maintained in seven glass aquaria (180 l capacity), in triplicate, with 10 fish in each aquarium. Fish were injected (intramuscular) each with 0.1 ml of *A. hydrophila* containing 10^3^, 10^4^, 10^5^, 10^6^, 10^7^, 10^8^ and 10^9^ and CFU/ml (the practice were performed by the permission of the Animal Ethics Committee, College of Fisheries, Mangalore). Control fish in tank eight received 0.1 ml PBS. Mortality was noted at different hours interval for 10 days. The pathogenicity (degree of virulence) were estimated as reported by Reed and Muench [37]. LD_50_ of *A. hydrophila* in pangasius was found to be 4.5 × 10^7^ CFU/ml (Table 1). At higher concentration (10^9^ CFU/ml) 90 % mortality was observed and there was no mortality within 10 d post injection at 10^3 CFU/ml^. No mortality was observed in fish injected with phosphate buffered saline. Therefore, 4.5 × 10^7^ CFU/ml, was considered for challenge studies.

**Table 1:** Details of LD_50_ of *Aeromonas hydrophila* in pangasius catfish.

**No. of Bacterial cell (CFU/ml)**	**Initial number**	**No. of fish dead**	**No. of fish survived**	**Death ratio**	**Survival ratio**	**Mortality rate**	**Cumulative mortality (%)**
10^9^	30	30	0	96	0	96/96	100
10^8^	30	27	3	66	3	66/69	95.65
10^7^	30	20	10	39	13	39/52	75
10^6^	30	11	19	19	32	19/51	37.19
10^5^	30	7	23	8	55	8/63	12.70
10^4^	30	1	29	1	84	1/85	1.18
10^3^	30	0	30	0	114	0/114	0.00

Proportionate distance = [(mortalityatdilutionnextabove50%−50%]/[mortalitynextabove50%)−(mortalitynextbelow50%]

Log LD_50_ = Dilution above 50% - (Proportionate distance × Dilution factor)

**LD_50_** = 4.5 × 10^7^ CFU/ml

#### 2.9.2 Vaccine efficacy determination by challenge study

Twenty six orally vaccinated *P. hypophthalmus* from each tank of vaccinated (BF and FC) and unvaccinated fishes were injected (IM) with 0.2 ml of 18 h old culture of *A. hydrophila* at 4.5 × 10^7 CFU/ml^. Manifestation of clinical signs, morbidity and mortality were viewed for 12 days. Fresh blood from moribund fish and streaking were done from kidney on RS media (Specific for *A. hydrophila* isolation) for the confirmation of fish death. Vaccine efficacy were measured by relative percent survival was recorded as described by Amend [38].

RPS={1−(%mortalityofvaccinatedgroup)/(%mortalityincontrol)}×100

### 2.10 Serum antibody response of *P. hypophthalmus*

#### 2.10.1 Purification of immunoglobulin

Pangasius IgM purification process in the present study were done according to Babu et al. [39] using a 5 ml BSA-CL agarose column. Extracted samples were merged and dialysis were done at 4 °C using a membrane. The membrane were transferred to the tray to condense and concentrated IgM was preserved at -40 °C for further analysis.

#### 2.10.2 Development of polyclonal antibody (PAb) against *P. hypophthalmus* IgM

Two healthy rabbits were reared with proper house for the development of polyclonal antibody. Serum were taken before injecting the purified IgM, considered as control. Rabbits were injected intramuscularly (IM) with 400 µl pure IgM (100 µgml^−1^) emulsified in Freund's complete Adjuvant (1:1). On days 14, 28, and 35, the remaining three booster doses were given with incomplete Freund's adjuvant. Anti-IgM rabbit antiserum that had been highly immunized was filtered through a 0.45 m syringe filter and purified on a Protein-A Sepharose column. The antiserum was separated into 0.5 ml aliquots and kept at -20 °C until it was used. During the rabbits' rearing, the College's Animal Ethics Committee's codal formalities were observed.

#### 2.10.3 Characterization of polyclonal antibodies

Pangasius immunoglobulins were characterized by 15% SDS-PAGE Laemmli [40]. Briefly, samples were mixed 1:1 with sample buffer, boiled for 5 min at 95 °C, and then placed onto the gel with the molecular weight markers. The dye front was allowed to reach the bottom of the gel by running it at a continuous voltage (80 V). The polyvinyldine difluoride (PVDF) membrane (0.2 µm, Amersham) was activated by soaking it in cooled methanol for 15 min, then washing it and equilibrating it separately in a Western blot buffer. Using a semi-dry-transblot device, proteins were transferred to PVDF membrane for 1 h at a steady 250 milliampere (mA) current and 25 volts (Chromous Biotech, Bangalore). PVDF protein lanes were cut into strips and reacted overnight with blocking solution (3% BSA-PBS). After that, the paper lanes were incubated with immune serum for 2 ½ h. Anti-rabbit IgG-HRP (1:2000) was produced in blocking buffer and incubated for 45 min at room temperature (RT) on a rocker shaker after three washes with PBS –Tween 20 (0.05%). Strips were washed three times with wash buffer before being incubated for 5 min with the substrate-chromogen complex (H2O2 – 3, 3′- Diaminobenzidine tetrahydrochloride hydrate, Sigma, USA) in a rocker shaker. The reaction was stopped by using wash buffer to clean the PVDF membrane strips. After the strips were properly dried, a brown band appeared, which was compared to a standard protein molecular weight marker (wide range, Sigma USA).

#### 2.10.4 Evaluation of serum antibody response by polyclonal antibody (PAb) based ELISA

The ELISA was performed as stated by Furuta et al. [41] with some modifications. *A. hydrophila* antigen (0.1 ml) was coated to Elisa plate wells (10^9^ cells). Free sites were block by coating the plate with 300 µl of PBS (0.05% tween and 1% milk powder) for 1 h. Poured off the caesin, plate were wash (3 × 5 min) with PBS tween 20. Serum samples were added (starting dilution 1:4) and incubated for 1 h at 37 °C. Plate were washed (3 × 5 min) with PBS tween 20 and one time with PBS. After that, 0.1 ml of anti- *P. hypophthalmus* IgM polyclonal antibody was added, which was incubated for 1 h at room temperature before being washed. The bound antibodies were washed after a 45 min reaction at room temperature with HRP conjugated DAKO goat anti mouse IgG (Sigma, USA) diluted 1:4000. The substrate (TMB) was added and incubated for 5 min before the reaction was halted by adding 50 µl/2N H2SO4 to each well. In an ELISA reader, the color developed was read at 450 nm (Bioteck, USA).

### 2.11 Analysis of haemato-biochemical parameters

Hematological parameters such as hemoglobin, hematocrit, erythrocyte count, mean corpuscular volume (MCV), mean cell hemoglobin (MCH), mean corpuscular hemoglobin concentration (MCHC), total leucocyte count (TLC), platelets were determined using automatic hematology analyzer (PCE-210) and biochemical parameters such as serum glutamate pyruvate transaminase (SGOPT), serum glutamate oxaloacetate transaminase (SGOT), alkaline phosphate (ALP), cholesterol, triglycerides, serum glucose, total protein, albumin, globulin, A:G ratio and total immunoglobulin were analyzed using kits (AGD Biomedicals Pvt. Ltd., Mumbai) by clinical chemistry analyzer (AGD-2020) at Animal Disease Diagnostic Laboratory and Information Centre (Institute of Animal Health and Veterinary Biologicals, Mangalore, Karnataka).

### 2.12 Histology of gut

For histological study, healthy fishes were euthanized and mid gut were separated, washed in normal saline and preserved in 10% neutral buffered formalin for 72 h for fixation. All the histological procedures were followed as detailed by Bullock [42]. The tissues on the glass slides were analysed through the light microscope (Olympus, BX3-25ND25, Japan) to observe the gut associated lymphoid tissue (GALT).

### 2.13 Statistical analysis

All statistical analysis was performed using IBM SPSS version 20 software. All of the results were analyzed using one-way analysis of variance (ANOVA) and Duncan's multiple range tests after being represented as means ±SE of three replications. Differences were determined and *P <* 0.05 was used to determine whether they were statistically significant.

## 3 Results

### 3.1 Characterization of biofilms through light microscopy and FE-SEM

**Fig. 1:**
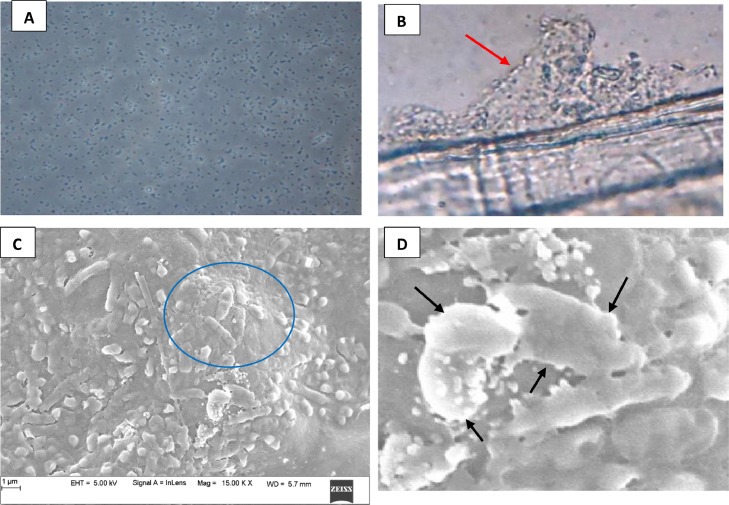
A: Free cells of *A. hydrophila* from the one day cultured broth (100X); B: Microphotograph of the 4-day old biofilm cell of *A. hydrophila,* note that the fingerlike projections (red arrow) of the glycocalyx (100X) C: FE-SEM of 4-day old biofilm cell of A*. hydrophila*. Note that the clumps of bacterial cell (circle) (15000X) D: Computer blown up image of biofilm of *A. hydrophila* showing the *A. hydrophila* inside the glycocalyx matrix (arrow).

Light microscopy and Field Emission Scanning Electron Microscopy (FE-SEM) revealed projection of biofilm and encapsulation of bacteria, saline features for biofilm bacteria (Fig. 1A–D).

### 3.2 Effects of *A. hydrophila* biofilm oral vaccine on growth of *P. hypophthalmus*

Significantly improved weight gain was achieved in BF fed fishes (46.91 ± 0.59 g) compared to FC (35.94 ± 0.21 g) and control group (34.92 ± 0.35 g) (*P <* 0.05). Significant lower but best FCR was observed in BF vaccine containing diet (1.190 ± 0.02) in comparison of FC (1.54 ± 0.01) and control diet (1.58 ± 0.03) is presented in Table 2 (*P <* 0.05).

**Table 2:** Growth and feed utilization of *P. hypophthalmus* recorded under vaccinated and control groups.

**Parameters**	**Treatments**		
	**BF**	**FC**	**Control**
Initial weight (g)	12.60* ± 0.15^a^	12.33 ± 0.33^a^	12.34 ± 0.32^a^
Final weight (g)	59.51 ± 0.62^a^	46.93 ± 1.74^b^	47.26 ± 0.64^b^
Weight gain (g)	46.91 ± 0.59^a^	35.94 ± 0.21^b^	34.92 ± 0.35^b^
% weight gain	372.32 ± 6.17^a^	291.80 ± 5.94^b^	283.21 ± 5.36^b^
Average daily gain (g/day)	0.59 ± 0.01^a^	0.45 ± 0.00^b^	0.44 ± 0.00^b^
Specific growth rate (% day ^−1^)	1.94 ± 0.02^a^	1.71 ± 0.02^b^	1.68 ± 0.02^b^
Feed conversion ratio (FCR)	1.19 ± 0.02^b^	1.54 ± 0.01^a^	1.58 ± 0.03^a^
Protein efficiency ratio (PER)	2.58 ± 0.07^a^	2.03 ± 0.02^b^	1.98 ± 0.03^b^
Feeding rate (FR)	1.97 ± 0.06^b^	2.15 ± 0.03^a^	2.25 ± 0.05^a^
Survival (%)	100 ± 0.00 ^a^	100 ± 0.00 ^a^	100 ± 0.00 ^a^

*Data are expressed as mean ± standard error (M ± SE). The values with different superscript letters in the same row are significantly different (*P <* 0.05)

Weight gain (g) = Mean final weight (g) – Mean initial weight (g)

% Weight gai*n =* [(Meaninitialweight−Meanfinalweight)/(Meaninitialweight)]×100

ADG (g/day) = (Finalweight−Initialweight)/Days

SGR (% day ^−1^) = [lnfinalweight−lninitialweight/Days]x100

FCR **=**(Dryweightofthefeedgiven(g)/(Gaininweightoffish(g))

PER= (Weightgain)/feedconsumed×percentageofproteininfeed

FR**=**(100×totalfeedintake)/([Days×(Initialbodyweighy+finalbodyweight)/2])

Survival (%) = [(Numberoffishesharvested)/(Numberoffishesstocked)]×100

### 3.3 Disease resistance against *A. hydrophila*

#### 3.3.1 Challenge with *A. hydrophila*

**Fig. 2:**
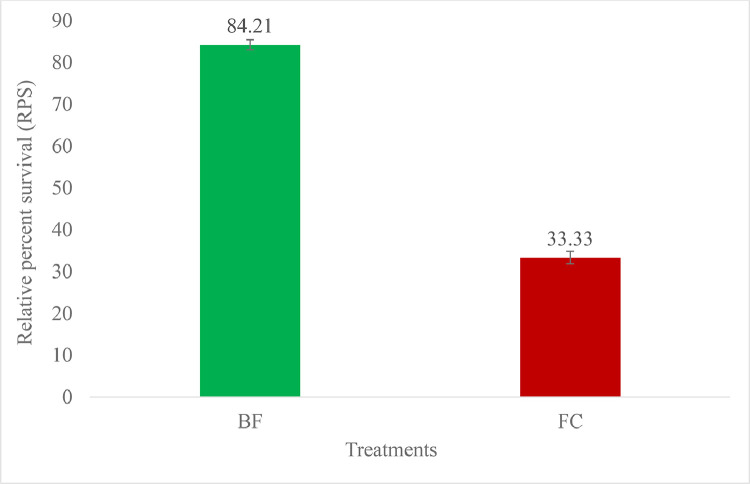
Relative percent survival of *P. hypophthalmus* upon challenge with *A. hydrophila*

Levels of protection with biofilm and free cell oral vaccination were assessed through homologous challenge with *A. hydrophila*. After 80 days (at 60 dpv) of the vaccine trial, fish from all the three groups including positive control were injected with 0.2 ml of bacterial suspension at 4.5 × 10^7^ CFU/ml. Fishes were injected with 0.2 ml PBS consider as negative control. Relative percent survival (Table 3, Fig. 2) in BF vaccinated group was higher (84.21 ± 1.49%) compared with that in FC (33.33 ± 1.21%). Several clinical signs has appeared in all treatments group including sluggish movement, tail and fins rot, haemorrhagic lesions on the dorsal peduncle, eyes and skin-muscles. Sloughed off epidermis, abrasion, abdominal distention and dropsy also observed in the challenged fishes. However, these clinical symptoms were apparently less in vaccinated fish. Moreover, the total fish mortality after challenge with *A. hydrophila* decrease significantly (*P <* 0.05) in BF vaccinated group compared to FC and control. *A. hydrophila* from blood and kidney of moribund fish were collected aseptically for the confirmation of the death.

**Table 3:** Protection level in vaccinated *P. hypophthalmus* challenged with *A. hydrophila* at 4.5 × 10^7^ CFU/ml.

**Treatments**	**No. of fish challenged**	**No. of fish survived**	**No. of fish dead**	**% cumulative mortality**	**% survival**	**RPS (%)**	**Independent T test**
Biofilm vaccine	78 ± 0.60	69 ± 0.60	9 ± 0.58	11.54 ± 0.63^c^	88.46 ± 2.10^a^	84.21 ± 1.49^a^	T< 0.05
Free cell vaccine	78 ± 0.58	40 ± 1.20	38 ± 1.20	48.72 ± 1.18^b^	51.28 ± 1.85^b^	33.33 ± 1.21^b^	
Control	78 ± 0.60	21 ± 1.20	57 ± 1.73	73.07 ± 1.88^a^	26.92 ± 0.71^c^	-	

Values are expressed as mean ± SE, in the columns with different superscripts differ significantly (*P <* 0.05).

### 3.4 Serum antibody response of *P. hypophthalmus*

#### 3.4.1 IgM purification and characterization

**Fig. 3:**
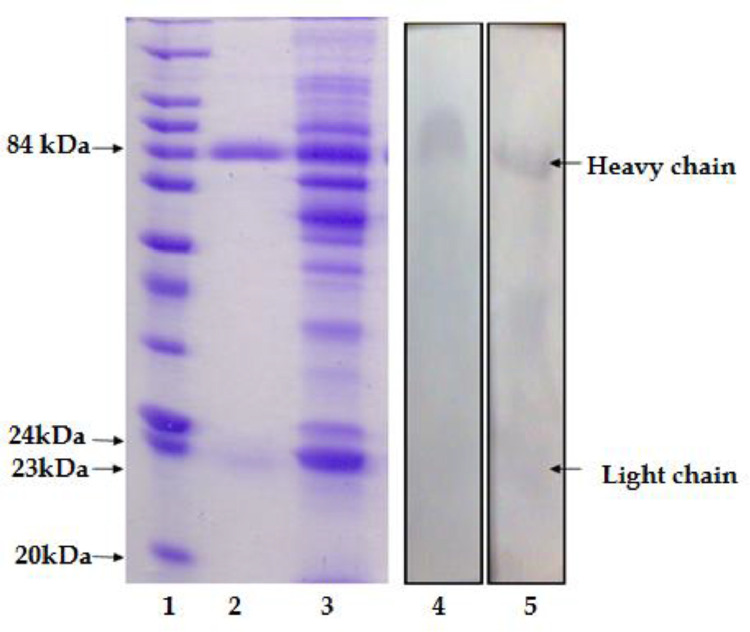
SDS – PAGE (A) and western blot (B) analysis of the purified pangasius immunoglobulin (Ig): Lane 1: Molecular weight marker Lane 2: Affinity purified serum, Lane 3: Whole serum of pangasius, Lane 4 and 5: western blots showing reactivity of the PAb to the heavy chain of the purified Ig.

The SDS-PAGE gel of extracted pangasius IgM displayed a 24 kDa band for the light chain (Fab) and a second 84 kDa band for the heavy chain (Fc) (Fig. 3).

#### 3.4.2 Measurements of serum antibody by polyclonal antibody (PAb) based ELISA

**Fig. 4:**
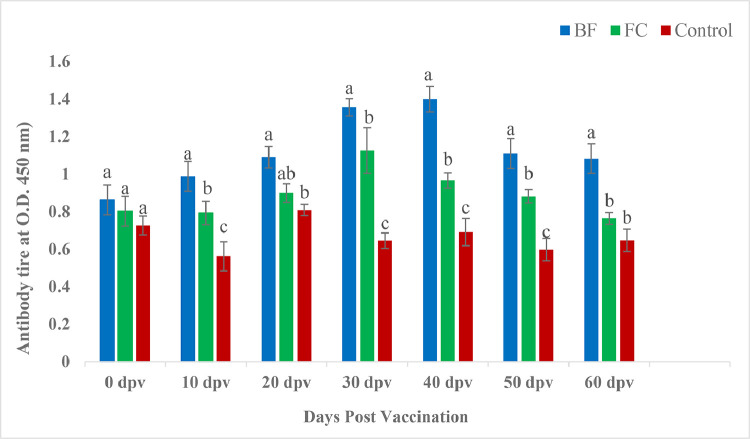
ELISA antibody titer in *P. hypophthalmus* orally vaccinated with BF and FC of *A. hydrophila*.

The serum antibody activity of the pangasius catfish respond strongly to vaccination. Serum antibody titer (two fold dilution) in ELISA of the treatments and control from 0th to 60th dpv, are depicted in Fig. 4. Higher antibody titer was observed in the BF group compared that with the FC and control groups.

### 3.5 Haematological parameters

Haematological profiles (TEC, Hb, PCV, TLC and Platelets) and indices (MCV, MCH, and MCHC) of vaccinated and unvaccinated *P. hypophthalmus* fingerlings are recorded in Table 4. The results exhibited that there were significant increase in total erythrocyte counts (TEC) in vaccinated fishes compared to control group on 0 day post vaccination (dpv). Moreover, TEC in biofilm vaccinated (BF) were significantly higher than FC vaccinated group on 0 dpv (*P <* 0.05). TEC were more excelled (*P <* 0.05) in BF treated fish compared to both vaccinated FC and control group, on 10 dpv. However BF group had the higher but no meaningful variations in total RBC counts at 30, 40, 50 and 60 dpv (Table 4). Higher haemoglobin were recorded in BF vaccinated fishes (Table 4). Significant increment of PCV was noted in BF vaccinated fishes compared to both vaccinated FC and unvaccinated fishes at 10 and 20 dpv. Mean cell volume (MCV) were found stable at 0, 10, 50 and 60 dpv in all the treatments. In general higher level of MCV were detected in BF treated fishes compared to both FC and control group, except 10, 20 and 60 dpv. The level of MCH and MCHC were higher at 0 to 10 dpv but no significant differences were noted. In general, except 10 dpv both MCH and MCHC were found similar among the treatments (P>0.05) (Table 4).

**Table 4:** Haematological profiles, total erythrocyte count (TEC), haemoglobin content (Hb), packed cell volume (PCV), mean cell volume (MCV), mean cell haemoglobin (MCH), mean cell haemoglobin concentration (MCHC), total leukocyte count (TLC) and Platelets of oral vaccinated and control *P. hypophthalmus*.

**Parameters**	**Treatments**	**0 dpv**	**10 dpv**	**20 dpv**	**30 dpv**	**40 dpv**	**50 dpv**	**60 dpv**
TEC (10^6^ mm^−3^)	BF	2.64 ± 0.05^a^	2.60 ± 0.13^a^	2.52 ± 0.14^a^	2.54 ± 0.12^a^	2.80 ± 0.09^a^	2.62 ± 0.10^a^	2.94 ± 0.09^a^
	FC	1.80 ± 0.05^b^	1.78 ± 0.09^b^	2.24 ± 0.04^ab^	2.48 ± 0.12^a^	2.65 ± 0.13^a^	2.34 ± 0.148^a^	2.63 ± 0.15^a^
	Control	1.79 ± 0.08^b^	1.82 ± 0.10^b^	2.18 ± 0.07^bc^	2.36 ± 0.11^a^	2.87 ± 0.07^a^	2.39 ± 0.12^a^	2.90 ± 0.05^a^
Hb (g dL^−1^)	BF	11.60 ± 0.15^a^	11.20 ± 0.37^a^	11.50 ± 0.21^a^	13.60 ± 0.37^a^	13.20 ± 0.14^a^	11.60 ± 1.55^a^	14.30 ± 0.55^a^
	FC	7.40 ± 0.46^b^	8.10 ± 0.57^b^	9.40 ± 1.04^a^	12.10 ± 0.84^a^	12.0 ± 0.68^a^	9.40 ± 0.58^a^	14.10 ± 0.86^a^
	Control	7.90 ± 0.17^b^	8.20 ± 1.04^b^	9.70 ± 1.44^a^	12.10 ± 0.31^a^	12.50 ± 1.79^a^	9.80 ± 0.23^a^	14.20 ± 0.51^a^
PCV (%)	BF	33.60 ± 1.21^a^	33.70 ± 1.21^a^	34.40 ± 0.46^a^	35.77 ± 2.26^a^	39.0 ± 2.19^a^	39.70 ± 2.70^a^	41.10 ± 2.10^a^
	FC	21.60 ± 0.57^b^	23.60 ± 2.25^b^	28.90 ± 038^a^	30.40 ± 3.11^a^	36.0 ± 0.57^a^	36.50 ± 0.57^a^	38.20 ± 1.21^a^
	Control	22.10 ± 2.36^b^	24.10 ± 0.57^b^	30.90 ± 2.67^a^	31.20 ± 1.09^a^	38.0 ± 1.15^a^	38.0 ± 4.04^a^	40.0 ± 5.84^a^
MCV (in fl)	BF	127.27 ± 2.73^a^	129.62 ± 1.15^a^	136.51 ± 1.21^a^	140.83 ± 2.79^a^	139.40 ± 1.5^a^	151.53 ± 2.30^a^	139.80 ± 2.42^a^
	FC	120.00 ± 2.30^a^	132.58 ± 3.53^a^	129.02 ± 1.15^b^	122.58 ± 1.73^b^	135.85 ± 0.57^ab^	155.98 ± 1.57^ab^	145.4 ± 1.78^a^
	Control	123.46 ± 2.62^a^	132.42 ± 1.08^a^	141.74 ± 1.10^c^	132.20 ± 2.17^c^	132.5 ± 1.15^bc^	158.99 ± 1.15^bc^	137.93 ± 4.25^a^
MCH (pg)	BF	43.94 ± 1.15^a^	43.08 ± 1.73^a^	46.0 ± 1.85^a^	53.54 ± 1.15^a^	47.14 ± 1.15^a^	44.27 ± 1.15^a^	48.64 ± 1.15^a^
	FC	41.11 ± 1.05^a^	44.94 ± 2.93^a^	41.96 ± 4.04^a^	48.79 ± 0.51^a^	45.28 ± 1.15^a^	39.17 ± 2.88^a^	53.61 ± 2.30^a^
	Control	44.13 ± 1.15^a^	45.05 ± 1.73^a^	44.49 ± 3.46^a^	51.27 ± 2.35^a^	43.55 ± 1.15^a^	39.04 ± 1.15^a^	48.96 ± 1.23^a^
MCHC (%)	BF	34.52 ± 1.15^a^	33.23 ± 1.73^a^	33.43 ± 1.15^a^	38.02 ± 1.15^a^	33.85 ± 1.15^a^	29.22 ± 1.15^a^	34.79 ± 1.73^a^
	FC	34.4 ± 2.30^a^	33.90 ± 0.61^a^	32.52 ± 1.73^a^	39.80 ± 1.73^a^	33.33 ± 0.57^a^	25.75 ± 1.15^a^	36.65 ± 2.30^a^
	Control	35.75 ± 2.88^a^	34.02 ± 0.94^a^	31.39 ± 2.30^a^	38.78 ± 1.15^a^	32.89 ± 2.3^a^	25.79 ± 1.15^a^	35.50 ± 1.73^a^
TLC (10^3^ mm^−3^)	BF	163.24 ± 2.30^a^	167 ± 1.73^ab^	167.8 ± 1.73^ac^	192 ± 1.15^a^	193.5 ± 1.15^a^	188.7 ± 3.17^a^	174.53 ± 2.30^a^
	FC	161.65 ± 1.15^a^	163.90 ± 2.30^bc^	156.9 ± 2.30^b^	173.1 ± 1.15^b^	169.5 ± 3.17^b^	168.1 ± 1.15^b^	162 ± 1.73^b^
	Control	159.47 ± 1.73^a^	161.30 ± 2.30^c^	161.3 ± 2.30^bc^	163 ± 1.73^c^	167.5 ± 0.57^b^	165 ± 1.73^b^	163 ± 2.30^b^
Platelets (10^3^ mm^−3^)	BF	72.09 ± 1.73^a^	62.67 ± 1.70^a^	49 ± 1.73^a^	66 ± 1.15^ab^	64 ± 1.73^ab^	87 ± 1.15^a^	87 ± 2.88^a^
	FC	73.09 ± 1.73^a^	59 ± 1.76^a^	42 ± 1.15^a^	62 ± 1.73^b^	60 ± 0.57^b^	62 ± 0.57^b^	61 ± 2.30^b^
	Control	76.26 ± 2.30^a^	72 ± 1.70^b^	51 ± 5.57^b^	70 ± 2.30^ac^	68.50 ± 1.15^ac^	60 ± 1.15^b^	60 ± 1.15^b^

Values are expressed as mean ± SE (*n =* 9) in the columns with different superscripts differ significantly (*p <* 0.05).

Total leukocyte count (TLC) plays an important role in defence mechanism. The level of TLC were significantly higher in BF group fishes on 40, 50 and 60 dpv compared with the FC and control group fishes. Despite the fact that vaccinated fish showed increased TLC, there was no noticeable difference between the treatments at 0 and 10 dpv. Platelets count were raised at 0 dpv in case of control fishes, moreover, significantly higher at 10 and 20 dpv than both the vaccinated treatments (*P <* 0.05). Surprisingly, at 50 and 60 dpv control fishes had significant lower platelets count compared to BF vaccinated fishes (Table 4).

### 3.6 Biochemical parameters

**Fig. 5:**
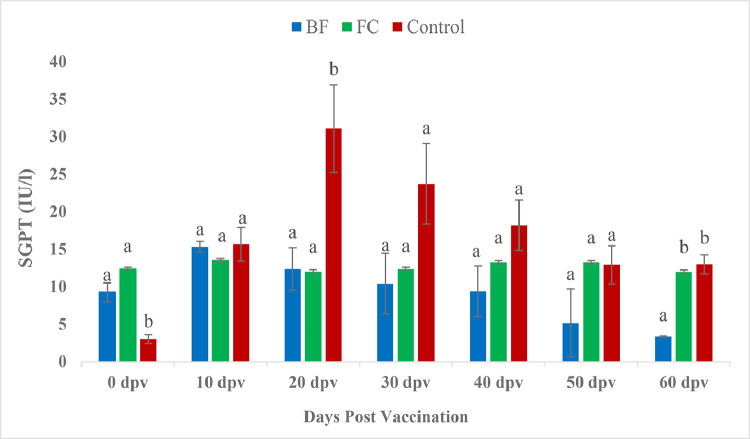
Comparison of SGPT (IU/l) between treatment groups.

**Fig. 6:**
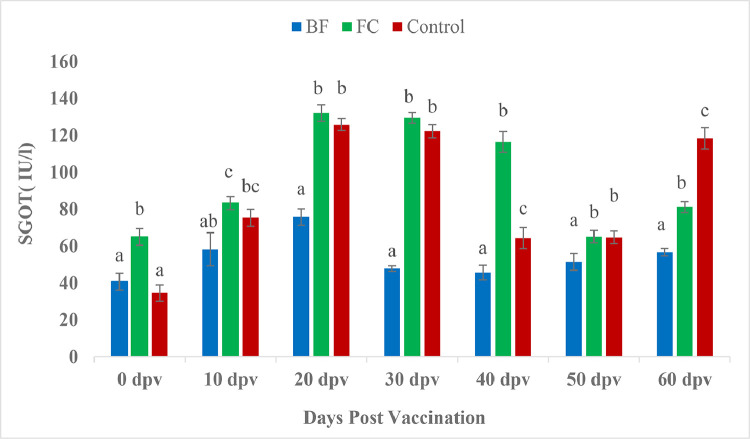
Comparison of SGOT (IU/l) between treatment groups. Same letters indicate no significant differences (P>0.05) between groups (Mean ± SE, *n =* 9).

**Fig. 7:**
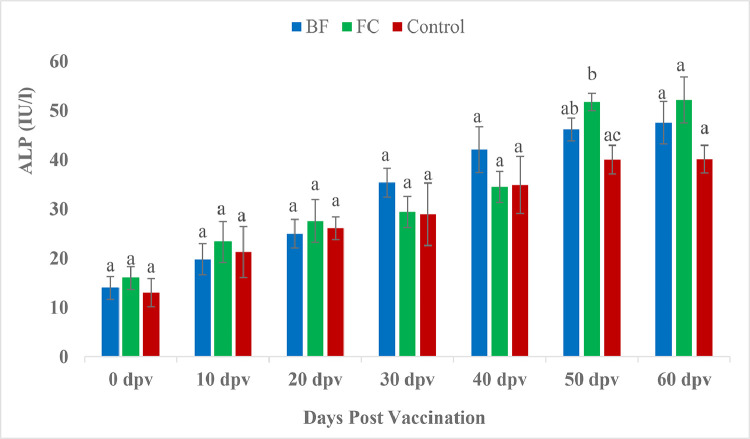
Comparison of alkaline phosphate (IU/l)) between treatment groups.

At 0 dpv, vaccinated groups showed significantly higher SGPT levels than the unvaccinated group (Fig. 5). In contrary, at 20 dpv, SGPT showed significant increase in control fishes than the vaccinated fishes. No significant difference noted among the treatments at 10, 30, 40 and 50 dpv (*P* > 0.05). However, at 60 dpv SGPT had reached significant lower level in BF vaccinated fishes compared to both FC vaccinated and control fishes. The SGOT level (Fig. 6) followed the same trend as SGPT in the in the present study. The ALP level in the FC marked a significant raise at 50 dpv, in comparison to the corresponding data for control (*P <* 0.05) (Fig. 7).

**Fig. 8:**
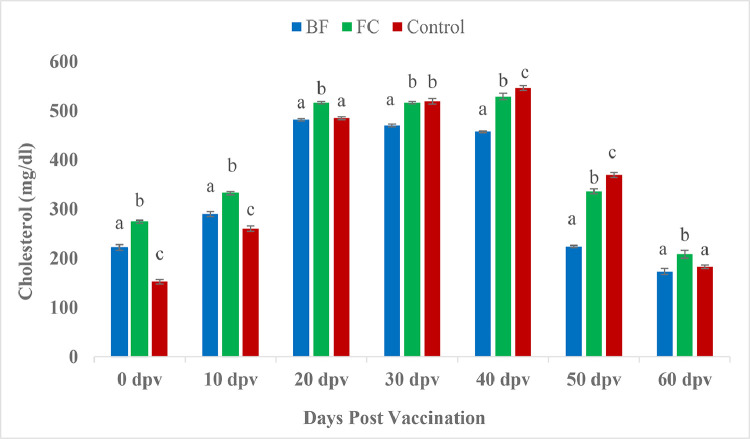
Comparison of cholesterol (mg/dl) between treatment groups.

**Fig. 9:**
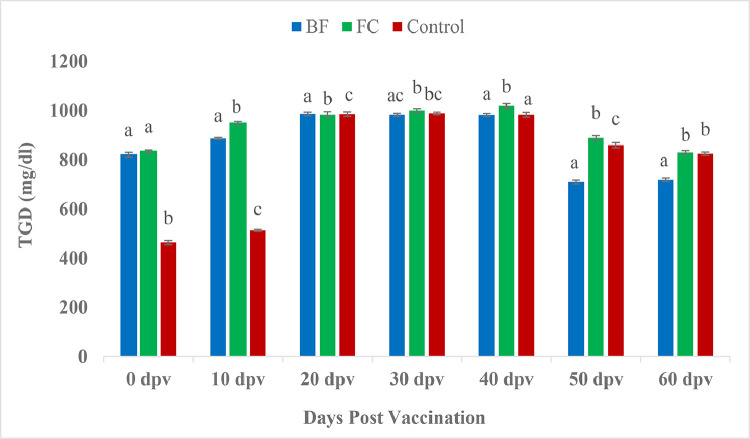
Comparison of triglycerides (mg/dl) between treatment groups. Same letters indicate no significant differences (*P* > 0.05) between groups (Mean ± SE, *n =* 9).

**Fig. 10:**
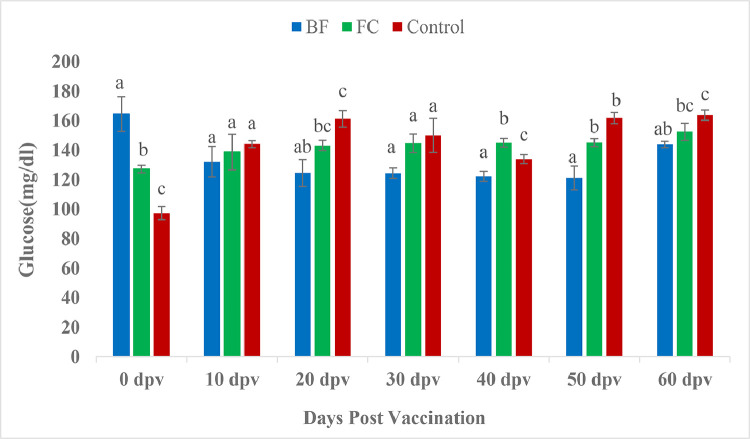
Comparison of glucose (mg/dl) between treatment groups. Different letters indicate significant differences (*P <* 0.05) between groups (Mean ± SE, *n =* 9).

The plasma cholesterol meaningfully onward in the vaccinated fishes than the unvaccinated control fishes in the beginning i.e. at 0 and 10 dpv. However, biofilm vaccinated fishes showed significant lower cholesterol level than control fishes by 30, 40 and 50 dpv (Fig. 8). Significantly higher serum triglyceride values were observed in vaccinated groups at 0 and 10 days than control group. However, at 60 dpv, biofilm vaccinated fishes registered significant lower triglyceride value in contrast with both FC and control group fishes (*P <* 0.05) (Fig. 9). Similar trend were also found in blood glucose level (Fig. 10).

Vaccinated BF group recorded a significant upward activity of total protein parallel to both the FC and control group throughout the study period except 20 dpv (*P <* 0.05) (Table 5). A level of albumin was significantly elevated 40 d post vaccination in the vaccinated group in comparison to the control group (Table 5). Oral vaccination of *A. hydrophila* antigens had strong respond to globulin on day 0, 20 and 40 post vaccination (*P <* 0.05). Yet, no higher deviation was recorded between vaccinated FC and unvaccinated control group at 10, 30, 50 and 60 dpv, while in vaccinated BF samples, globulins level stayed significantly higher on those days (Table 5).

**Table 5:** Biochemical parameters, total protein, albumin, globulin, A:G ratio and total immunoglobulin of oral vaccinated and control *P. hypophthalmus*.

**Parameters**	**Treatments**	**0 dpv**	**10 dpv**	**20 dpv**	**30 dpv**	**40 dpv**	**50 dpv**	**60 dpv**
Total protein (g/dl)	BF	5.19 ± 1.27^a^	5.72 ± 1.01^a^	5.61 ± 0.96^a^	6.93 ± 0.67^a^	6.90 ± 0.65^a^	5.97 ± 0.24^a^	5.37 ± 0.69^a^
	FC	3.92 ± 1.15^b^	3.79 ± 1.39^b^	3.80 ± 0.98^a^	4.07 ± 0.68^b^	4.56 ± 0.78^b^	3.34 ± 1.14^b^	3.91 ± 0.66^b^
	Control	2.97 ± 1.02^c^	3.73 ± 0.75^b^	4.67 ± 0.52^a^	3.85 ± 0.83^b^	3.87 ± 0.91^c^	3.43 ± 0.65^b^	3.85 ± 0.34^b^
Albumin (g/dl)	BF	0.44 ± 0.58^a^	0.54 ± 0.01^a^	0.59 ± 0.05^a^	0.82 ± 0.04^a^	1.11 ± 0.11^a^	0.65 ± 0.06^a^	0.51 ± 0.04^a^
	FC	0.42 ± 0.12^a^	0.53 ± 0.02^a^	0.57 ± 0.16^a^	0.75 ± 0.06^a^	1.02 ± 0.08^a^	0.55 ± 0.06^a^	0.47 ± 0.02^a^
	Control	0.43 ± 0.07^a^	0.51 ± 0.06^a^	0.53 ± 0.07^a^	0.79 ± 0.1^a^	0.68 ± 0.10^b^	0.56 ± 0.01^a^	0.41 ± 0.05^a^
Globulin (g/dl)	BF	4.75 ± 0.12^a^	5.18 ± 0.10^a^	4.14 ± 0.03^a^	6.11 ± 0.11^a^	5.79 ± 0.12^a^	5.32 ± 0.17^a^	4.86 ± 0.08^a^
	FC	3.50 ± 0.23^b^	3.26 ± 0.15^b^	3.23 ± 0.12^b^	3.32 ± 0.17^b^	3.54 ± 0.11^b^	2.87 ± 0.12^b^	3.44 ± 0.11^b^
	Control	2.54 ± 0.12^c^	3.22 ± 0.12^b^	2.02 ± 0.06^c^	3.06 ± 0.02^b^	3.19 ± 0.06^c^	2.79 ± 0.08^b^	3.44 ± 0.12^b^
A:G ratio	BF	0.09 ± 0.00^c^	0.10 ± 0.01^b^	0.12 ± 0.01^b^	0.13 ± 0.01^b^	0.19 ± 0.01^c^	0.12 ± 0.01^b^	0.11 ± 0.02^b^
	FC	0.12 ± 0.00^b^	0.16 ± 0.02^a^	0.18 ± 0.02^a^	0.23 ± 0.02^a^	0.21 ± 0.01^b^	0.20 ± 0.01^a^	0.14 ± 0.01^a^
	Control	0.17 ± 0.01^a^	0.16 ± 0.02^a^	0.13 ± 0.01^b^	0.26 ± 0.03^a^	0.29 ± 0.01^a^	0.20 ± 0.01^a^	0.12 ± 0.02^ab^
Total Immunoglobulin (g/dl)	BF	1.55 ± 0.30^a^	1.89 ± 0.18^a^	2.18 ± 0.30^a^	2.22 ± 0.33^a^	2.86 ± 0.22^a^	1.85 ± 0.15^a^	1.65 ± 0.25^a^
	FC	1.35 ± 0.07^b^	1.76 ± 0.08^a^	1.63 ± 0.22^b^	1.33 ± 0.35^b^	1.15 ± 0.5^b^	0.97 ± 0.13^b^	1.27 ± 0.25^a^
	Control	0.71 ± 0.05^b^	0.75 ± 0.15^b^	1.04 ± 0.25^c^	1.10 ± 0.07^b^	0.75 ± 0.35^b^	1.19 ± 0.05^b^	1.25 ± 0.12^a^

Values are expressed as mean ± SE (*n =* 9) in the columns with different superscripts differ significantly (*P <* 0.05)

Highest A/G ratio was registered in the unvaccinated fishes compared to vaccinated groups at 0, 10, 30 and 40 dpv, whereas least values were recorded in the BF vaccinated group (*P <* 0.05). Significantly elevated total immunoglobulin (Ig) activity were found in the BF, and FC groups compared to the control group. However, BF group was exhibited meaningfully increased (*P <* 0.05) Ig level than that of FC and unvaccinated group throughout the study period (Table 5).

### 3.7 Validation of gut associated lymphoid tissues (GALT)

**Fig. 11:**
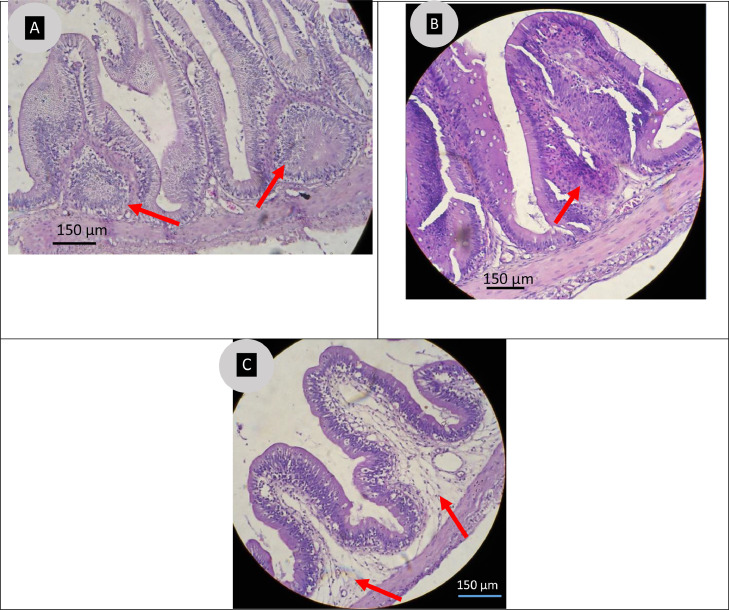
Histomorphological analysis of mid gut of *P. hypophthalmus* fed vaccinated and unvaccinated diets. **A:** Transvers section of BF vaccinated fishes showing two focal aggregations of lymphoid tissue (GALT) in lamina propria **B:** Gut histology of FC vaccinated fishes showing less aggregation of lymphoid cells near the lamina propria (red arrow) and **C:** Unvaccinated fishes showing no aggregation of lymphoid tissue (GALT) in either lamina propria or mucosal tissues (arrows) as compared with BF and FC group fishes.

Transvers section of mid gut in biofilm (BF) vaccinated fishes showed higher aggregation of GALT (Fig. 11 A). Moreover, FC group fishes showed less aggregation of lymphoid cells (Fig. 11 B). Lymphoid cells aggregation was not detected in the intestinal part of mid gut of the control group fishes (Fig. 11 C).

## 4 Discussion

The goal of this research was to assess the efficacy of oral immunization with biofilm of *Aeromonas hydrophila* vaccine on growth, haemato-biochemical and gut morphology of striped catfish, *P. hypophthalmus*.

### 4.1 Effect of biofilm oral vaccine on growth of pangasius juveniles

Present study successfully demonstrated growth-related effects in pangasius catfish vaccinated with *A. hydrophila* biofilm based oral vaccine. The study indicates that vaccination had positive effects on the growth of pangasius catfish. Biofilm fed fishes displayed significantly higher weight gain and decreased FCR (*p <* 0.05) (Table 2). Identical results compared to present studies were observed in ornamental goldfish, recorded highest weight gain at 25 dpv and 50 dpv in biofilm plus immunoadjuvant group fishes [43]. Oral route of vaccination considered as stress-free administration can play vital role in protecting a bacterial [44] and viral disease [45] suggested that the oral immunization had no effect on healthy fish growth. The non-stressful method of immunization described by Chair et al. 46] using encapsulated *V. anguillarum* bacterin within *Artemia nauplii* increased weight gain and feed conversion in European sea bass (*Dicentrarchus labrax*), which is also evident in the current investigation. The elevated growth in BF fishes might be due to the polymetric extracellular matrix of biofilm of *A. hydrophila*. A bacterial biofilm is a self-contained population of bacterium cells through extracellular polymeric substances (EPS) [47] that would facilitate the nutrient uptake [48]. Biofilms are bioactive substances and dietary stimulants that can help aquaculture organisms grow faster [49]. African catfish, *Clarias batrachus* vaccinated with feed based *A. hydrophila* GPl-04 vaccine did not adversely affect the growth [50]. Earlier studies were conducted with the employing of natural substrate based biofilm, achieved higher biomass compare to control at the end of the experiments [[51], [52], [53]].

### 4.2 Immune response and diseases resistance upon biofilm oral vaccine

An onward level of protection was registered in biofilm vaccinated pangasius in comparison with vaccinated FC and control fishes when injected with homologous intramuscular (i.m.) challenge (Table 3). Survival was significantly raised in BF vaccinated group (88.46 ± 2.10%) compared to both vaccinated FC group (51.28 ± 1.85%) and control group (26.92 ± 0.71%). As a result, the biofilm vaccine is thought to be more effective than other treatments since the relative percent survival (RPS) was 84.21 ± 1.49 % which is notably soaring than free cell vaccine (*P <* 0.05) (33.33 ± 1.21%) (Fig. 2). The present study are in accordance with previous authors who reported similar findings with biofilm vaccine in Indian major carps [[25], [26], [27]], Tilapia [28,54] gold fish [55] and walking catfish [26]. A higher protection level (85.4%) was achieved through the oral vaccine against *V. anguillarum* biofilm was compared to the free cell vaccine (27%) in Asian seabass, *Lates calcarifer* [29]. Findings of the present study closely agree with the similar study conducted in Taiwan [56,57]. They had reported significantly higher RPS in biofilm vaccines against *Lactococcus garvieae* infection in Mullet (77%) and in *Photobacterium damselae* infection in giant grouper (62%). Moreover, cross protection of different geographical *A. hydrophila* isolates were tested and found meaningful RPS (*p <* 0.05) rate of 81.5% in the vaccinated fish compared to unvaccinated fish (21%) [58]. The better protection of biofilm in oral vaccination could be attributed to the nature of glycocalyx, which protects vaccine antigen from digestion in the stomach, as demonstrated in the current work.

### 4.3 Antibody titre of Polyclonal antibody based ELISA

The biofilm vaccinated *P. hypophthalmus* had higher antibody titre than those vaccinated with free cell on all days of post vaccination except 0 and 20 dpv. On 0th dpv, the antibody titer was similar to all the experimental groups. The presence of natural antibodies in the fish against the widespread *A. hydrophila* could explain the high level of antibodies found in the unvaccinated control group. Previous research have also reported similar observations by antibody-agglutination titre method [25,26,28] as well as in MAb based ELISA [27,58,59,]. Better performance with higher antibody titer in BF groups than in FC and control groups, may be attributed to biofilm glycocalyx [60] that protect the *A. hydrophila* antigens from acid degradation in the stomach of pangasius could facilitates the bunch of antigens to the posterior gut to be traced and processed by the memory cells, and finally, the immune response could activated [18].

### 4.4 Effect of haemato-biochemical parameters

A range of blood parameters were evaluated to assess the effect of vaccination on fish [59,61]. In the present study significant increase in TEC in BF vaccinated fishes compared to both vaccinated FC and unvaccinated control group on 0 and 10 day post vaccination (dpv) were observed. Furthermore, on 20 dpv, significant higher TEC were noted on BF fed fishes than the basal diet fed fishes (Table 4). Similarly, significantly higher erythrocytes count were reported on day 21 in orally vaccinated Nile tilapia [61]. Red blood cell (RBC) count were 0.86 (× 10^6^ mm^–3^) at 25 dpv in the control group, and this significantly increased to 1.35 (x10^6^ mm^–3^) with biofilm plus adjuvant (BF2) group fishes (*P <* 0.05) [43]. At 14 days after vaccination against *A. hydrophila*, Irianto et al. [55] noticed an increase in erythrocyte count in goldfish (*C. auratus*). Studies have shown that a decrease in the number of erythrocytes in the blood as well as a decrease in the hematocrit percentage can be indicators of bacterial infection [62,63]. Nile tilapia (*Oreochromis niloticus*) naturally invaded with *E. tarda* reported significant reduction in erythrocytes and haemoglobin level [62]. The total erythrocyte count, haemoglobin content were significantly enhanced in pangasius catfish fed dietary carotenoid supplementation diet [64] and biofilm vaccinated gold fish [43]. Immunostimulants have the ability to enhance RBC and haemoglobin levels in general, which could have been induced by biofilm supplementation in this study.

Haematological indices including PCV, MCV, MCH and MCHC can be regarded as appropriate indicators to find out possible physiological response in fish. Current experiment showed elevated level of hematocrit (PCV) and MCV in BF group in comparison to both free cell and control groups at different timeline. The level of MCH and MCHC in the current work were higher but no remarkable changes were noted in all the experimental groups except 10 dpv. The past study showed significantly higher hematocrit percentage from orally vaccinated fish than in the unvaccinated control fish Silva et al. 61].

Total leukocyte counts (TLC) plays an important role in defence mechanism. On day 30 after vaccination, the TLC was markedly higher in BF group than that of both FC and control group, and it reached a maximum value of 193.5 ± 1.15 (x 10^3^ mm^–3^) on day 40 dpv but decreasing trend at 50 and 60 dpv although level of TLC significantly higher than other groups. The lower level of TLC in FC group in this study might be due to the degradation of antigen in acid stomach of fish, is also evident as GALT was less aggregated in FC vaccinated fish. The findings are in accordance with Silva et al. in 2009 [61] and Sirimanapong et al. in 2014 [59], noted significant higher leukocyte counts over the course of the research. Biofilm fed fishes in the present study showed increased WBCs might be due to the improved general health condition by better immune responses and various actions of the specific cell-mediated immune systems. Result of the present study correlated with the findings of the research of Monir et al. [65] and Rahman et al. [66]. In their studies, they found that the leukocyte, lymphocytes, monocytes, granulocytes counts were boosted substantially (*p <* 0.05) by the feed-based bi-valent vaccines in red tilapia and catfish respectively. Thrombocytes counts were significantly higher in BF group fishes at 50 and 60 dpv. Silva et al. 61] reported similar results in the orally immunized tilapia where number of thrombocytes were up regulated to 40.78 (x 10^3^ mm^−1^) than the control {31.11 (x 10^3^ mm^−1^)} on day 21 after vaccination.

The evaluation and profiling of serum biochemical characteristics is thought to be a useful technique in clinical pathology for detecting tissue or organ damage, disease progression, and making appropriate medical decisions [67,68]. Serum glutamate pyruvate transaminase (SGPT) and serum glutamate oxaloacetate transaminase (SGOT) activities are associated with hepatic pathology [69]. In the present study, significantly elevated SGPT was recorded in vaccinated groups in comparison to the control group at 0 dpv. However, at 20 dpv, SGPT level were significantly decrease in both the vaccinated groups over control. Moreover, BF group had down regulated (*P <* 0.05) SGPT level compared to both FC and control group on 60 dpv indicating normal health status of biofilm vaccinated fishes. Similarly, SGOT level in the present study followed the same trend of SGPT. *Aeromonas* infection in goldfish [70] and rainbow trout [71] resulted increased SGPT and SGOT. Previous reports showed elevated SGPT and SGOT following monogenean infestation [72] and toxicological challenge in *Salmo gairdneri* [73] and *Parophrys vetulus* [69]. A significant increase in the SGPT and SGOT levels is caused by any acute injuries or necrotic lesions in the liver [67,74]. The severity of histopathology in the liver, gut, and trunk kidney was linked positively with SGPT and SGOT, according to Chen et al. [67]. The histopathological findings in the present study, in BF fed *P. hypophthalmus*, showed normal architecture however, several histopathological abnormalities were detected in gills, liver and kidney of FC and control group fishes [75]. Our results also corroborate the finding of Biswas et al. [74], where extensive degenerated and necrotized head kidney tissues were observed in all the non-vaccinated fish, *L. rohita* against *A. hydrophila* vaccine. The ALP activity of the pangasius catfish did not respond strongly to vaccination except on 50 dpv (Fig. 7). But the levels of serum enzymes including SGPT, SGOT and ALP in BF vaccinated fishes were lower than other treatments. The decline in such blood enzymes in the present study could explain that the extracellular polymeric substances of biofilm of *A. hydrophila* have the potential to benefit the health of fish by improving liver function.

The concentration of serum glucose and triglycerides is commonly utilized as a health indicator in fish [76]. The plasma cholesterol, triglycerides and glucose level significantly increased in the vaccinated fishes than the unvaccinated control fishes in the beginning of the analysed time point however at the end of the trial, biofilm (BF) vaccinated fishes showed significant lower activity. This might be due to the capability of biofilm of *A. hydrophila* to reduce the effect of stressors. Similar observation were noticed in pangasius catfish fed with fucoidan rich sea weed extract [77]. Herbal extracts are well known suitable immunostimulants to boost the non-specific immune system as well as to keep the aquaculture organisms stress free [78]. Rainbow trout fed with both heat killed and freeze dried probiotic bacteria, *L. rhamnosus* at 10^11^CFU g^−1^ had significant upregulated cholesterol and triglycerides level [79].

The presence of significant total protein, albumin, and globulin levels in the serum proteins suggests that high amounts are likely due to the improvement of the non-specific immune response of fish [67,80]. In the present study, at 40 dpv the serum albumin activity of both vaccinated groups were reached a maximum value of 1.11 ± 0.11 (g/dl) and 1.02 ± 0.08 (g/dl) for BF and FC respectively than that of the control (0.68 ± 0.10 (g/dl) (Table 5). Compared to other treatments vaccinated BF group documented a notably (*P <* 0.05) higher activity of total protein for any time points analysed and reached at peak at 30 dpv (6.93 ± 0.67 g/dl). The finding in the present study are in agreement of Viji et al. [43] reported higher albumin level in biofilm groups than that of control group. An increase in albumin and globulin levels, as well as a drop in the A/G ratio, is thought to indicate a strong innate reaction in fish [72]. In the present study, an increment in globulin content and the decrement in A/G ratio were found in BF vaccinated group. It was observed that globulin level was increased steadily with BF vaccinated fishes and peaked at 40 dpv (6.11 ± 0.11 g/dl) indicating increased phagocytic and leukocytes counts in this experiment (Table 5). The present study's findings are in line with Prabu et al. [77] showed significant higher globulin (6.28 g/dl) and lower A/G ratio (0.24 g/dl) in striped catfish, *P. hypophthalmus* fed with 3% fucoidan rich sea weed extract (FRSE) diet. Earlier studies demonstrated in an increment of total protein, albumin, globulin and decreased A/G ratio in different finfishes [43,81]. In general, BF group was exhibited higher albumin, total serum protein and total immunoglobulins than that of FC and control group throughout the study period.

### 4.5 Validation of gut associate lymphoid tissue

Oral vaccination has many advantages in addition to the ability to stimulate both adaptive and local responses [82]. The gut-associated lymphoid tissue (GALT) of vertebrates plays an important role in eliciting both systemic and mucosal immune response [28,83]. Earlier studies demonstrated GALT especially in second segment of carp [84] and Nile tilapia [28,54]. Present study, for the first time demonstrating the GALT in pangasius catfish employing biofilm of *A. hydrophila* oral vaccine (Fig. 11 A). Findings of the present study closely agree with a similar study conducted in Malaysia [85]. They found higher level of GALT development and lymphocytes association in the hindgut of vaccinated red hybrid tilapia, while those remain unchanged in the tissue of unvaccinated control group. In another study Kahieshesfandiari et al. [28] experimented biofilm vaccine of *S. agalactiae* in protecting tilapia from streptococcosis and demonstrated GALT in the intestine of Nile tilapia. Moreover, present study has validated the presence of GALT in mid gut of BF treated pangasius even after 60 dpv compared to 2 weeks post vaccination (wpv) in tilapia, an indication of longer retention of antigens in the gut. The second gut segment has approximately four times the number of intraepithelial macrophages (per enterocyte) as the first [83]. Antigens may be transferred from the second segment's enterocytes to lymphoid cells (B and T), macrophages, and dendritic cells, causing them to respond correctly [18,84]. Similarly, an increase in blood lymphocytes is likely due to the oral vaccine activating mucosal immunity in the gut's lymphoid tissues. The current investigation found anti- *A. hydrophila* antibodies in pangasius blood serum, which is consistent with the notion that the GATL in teleost fish can stimulate both local and systemic humoral immune responses. However, such claim requires further investigation on whole cell biofilm oral vaccine on striped catfish.

## 5 Conclusions

The findings suggest that present research approaches to fish health, such as nutritional, haematological, biochemical, immunological, and gut associate lymphoid tissue, could be useful in determining the effects of oral vaccines on fish. The present study indicated that feeding with biofilm vaccine significantly enhanced the pangasius growth. Importantly, the oral administration of *A. hydrophila* biofilm vaccination enhanced both non-specific humoral and cellular immune responses in pangasius, as measured in this study. Furthermore, specific immunoglobulin (IgM) level measured by polyclonal antibody was also increased by oral administration biofilm of *A. hydrophila* vaccines in parallel with the better health condition of *P. hypophthalmus*. Intestinal gut of the biofilm vaccinated fishes expressed GALT even at 60 dpv, convincingly confirming the presence of antigens in the posterior gut. All of these findings suggested that biofilm vaccine could be one of the promising vaccines for *A. hydrophila* infection in pangasius.

## Funding source

The first two authors warmly recognize the Indian Council of Agricultural Research for sponsoring their Ph.D. through the Netaji Subhas-ICAR International Fellowship 2015-16.

## Declaration of Competing Interest

There are no competing interests stated by the authors.

## Data Availability

Data will be made available on request.
